# Prognostic impact of atrial fibrillation in patients with severe acute respiratory syndrome coronavirus 2 infection

**DOI:** 10.1097/MD.0000000000026993

**Published:** 2021-08-20

**Authors:** Ji Hyun Lee, You Mi Hwang, Youngjin Cho, Il-Young Oh

**Affiliations:** a Cardiovascular Center, Seoul National University Bundang Hospital, Seoul National University College of Medicine, Seongnam, Korea; b Division of Cardiology, St. Vincent's Hospital, Department of Internal Medicine, the Catholic University of Korea, Seoul, Korea.

**Keywords:** atrial fibrillation, coronavirus disease-19, prognosis

## Abstract

The prognostic impact of atrial fibrillation (AF) in patients with severe acute respiratory syndrome coronavirus (SARS-CoV-2) infection has not been well evaluated. We estimated the prognostic implications of AF in SARS-CoV-2 confirmed patients.

The OpenData4Covid19 (https://hira-covid19.net) project is a global research collaboration on coronavirus disease (COVID-19), hosted by the Ministry of Health and Welfare of Korea and the Health Insurance Review and Assessment Service of Korea. This dataset comprises all COVID-19-tested patients and their individual histories of medical service use from January 1, 2017 to May 15, 2020. All patients >19 years with confirmed SARS-CoV-2 infection were included. The primary endpoint was a composite of death and intensive care unit admission.

In total, 7162 adults with SARS-CoV-2 infection were included in this study. The prevalence of AF was 1.8% (n = 130). Patients with AF had unfavorable characteristics, such as older age and higher prevalence of comorbidities. The primary endpoint was more common in patients with AF than in those without (33.9% vs 12.9%, *P* < .001). In the multivariable model, age (odds ratio [OR]: 1.035, 95% confidence interval [CI]: 1.030–1.040), female sex (OR: 0.618, 95% CI: 0.535–0.713), diabetes (OR: 1.341, 95% CI: 1.093–1.580), and chronic kidney disease (OR: 2.714, 95% CI: 1.541–4.777) were associated with the primary endpoint. However, AF was not an independent predictor of the primary endpoint (OR: 1.402, 95% CI: 0.932–2.108).

Patients with AF and concomitant SARS-CoV-2 infection had more comorbidities and a worse prognosis. However, an independent association between AF and adverse clinical outcomes was not evident.

## 1 Introduction

The coronavirus disease (COVID-19) pandemic has been spreading rapidly. The world is currently facing a critical challenge to public healthcare systems and struggling to the stop spread of the disease. Although infection with severe acute respiratory syndrome coronavirus (SARS-CoV-2) is often asymptomatic, severe acute respiratory distress leading to death may develop in some patients.^[1]^ Patients with concomitant cardiovascular disease are at a particularly higher risk of mortality.^[2,3]^

Atrial fibrillation (AF) is the most common sustained arrhythmia. The prevalence varies across different age groups; but, in 2015, the overall prevalence in Korea was estimated at about 1.5%.^[4]^ AF is associated with a negative prognostic impact, including a 1.5 to 2 fold increased risk of all-cause, and is associated with increased morbidity, such as heart failure (HF) and stroke.^[5]^ To date, there are sparse data regarding how AF influences the prognosis of SARS-CoV-2 infection. Thus, we aimed to investigate the prognostic impact of AF in patients with SARS-CoV-2 infection.

## 2 Materials and methods

We used the dataset from the OpenData4Covid19 (https://hira-covid19.net) project, a global research collaboration on COVID-19 hosted by the Ministry of Health and Welfare of Korea, and the Health Insurance Review and Assessment Service of Korea. This dataset, based on the insurance claims sent to health insurance review and assessment service from January to May 15th, 2020, includes all COVID-19-tested patients and their history of medical service use for the past 3 years (since January 2017) in Korea. SARS-CoV-2 infection was confirmed by real time-polymerase chain reaction (RT-PCR) analysis of a nasopharyngeal sample. These claims data include comprehensive information regarding the patients’ diagnosis and treatment. All patients with confirmed SARS-CoV-2 infection must be hospitalized in isolation rooms or quarantined in living treatment centers, according to their disease severity, until negative conversion of the RT-PCR COVID-19-test. A total of 234,427 patients who were tested for COVID-19 were included in this data set, and the individual information of the SARS-CoV-2 test results and mortality were also provided.

The first confirmed case of SARS-CoV-2 infection in South Korea was on January 19, 2020. Since then, all consecutive patients older than 19 years with confirmed SARS-CoV-2 infection were included in this study. AF and other comorbidities were defined using the international classification of disease, tenth revision, clinical modification codes (see Table, Supplemental Digital Content S1, which describes the definition of AF and comorbidities). In brief, AF was defined if the patients had any relevant international classification of disease, tenth revision, clinical modification codes (I480-I484, I489) from January 2017 to May 15, 2020 in the claims database. We classified the study population into 2 groups according to the presence or absence of AF. We subsequently compared the clinical course after confirmation of SARS-CoV-2 infection between the 2 groups.

The primary endpoint was a composite of mortality and intensive care unit (ICU) admission. The secondary endpoints included mortality, ICU admission, mechanical ventilator therapy (MV), and a composite of MV and high flow nasal cannula oxygen therapy. Detailed information on the claim codes for the definition of each endpoint is described in Supplemental Digital Content, Table S2. This study was approved by the institutional review board (approval number: X-2006–616–902) and conducted according to the principles of the Declaration of Helsinki. The requirement for informed consent was waived because of the minimal risk to participants.

### 2.1 Statistical analysis

Categorical variables are presented as numbers and frequencies, whereas continuous variables are presented as means ± standard deviations. Student *t* test was used to compare continuous variables and the Chi-Squared test was used to compare categorical variables.

Binary logistic regression analysis was used for estimating the risk of the study endpoints. The variables included in the multivariable model were age, sex, hypertension, diabetes, prior myocardial infarction, HF, stroke, chronic kidney disease, and chronic obstructive pulmonary disease. All statistical tests were performed with SAS version 9.4 (SAS Institute; Cary, NC), and *P* < .05 was considered statistically significant.

## 3 Results

A total of 7162 adults with confirmed SARS-CoV-2 infection were included in this study. Among the study population, 130 patients had AF. The baseline characteristics of the study population are provided in Table 1. Compared to non-AF patients, AF patients were older (71.9 ± 13.7 vs 47.3 ± 18.5 years, *P* < .001) and more likely to be male (53.9% vs 39.1%, *P* = .001). Regarding their history, AF patients had a higher prevalence of hypertension (75.4% vs 21.5%, *P* < .001), diabetes (35.4% vs 13.7%, *P* < .001), chronic kidney disease (3.1% vs 0.74%, *P* < .003), and chronic obstructive pulmonary disease (3.9% vs 0.84%, *P* < .001). AF patients were also significantly were more likely to have a history of cardiovascular diseases, including, coronary artery disease (30.0% vs 3.3%, *P* < .001), myocardial infarction (4.6% vs 0.5%, *P* < .001), heart failure (36.9% vs 1.6%, *P* < .001), stroke (17.7% vs 2.5%, *P* < .001), and peripheral artery disease (12.3% vs 6.3%, *P* = .005).

**Table 1:** Baseline characteristics of the study population.

	No AF n = 7032	AF n = 130	*P* value
Age	47.3 ± 18.5	71.9 ± 13.7	<.001
Male	2799 (39.1)	70 (53.9)	.001
Patient history			
Hypertension	1511 (21.5)	98 (75.4)	<.001
Diabetes	962 (13.7)	46 (35.4)	<.001
Coronary artery disease	232 (3.3)	39 (30.0)	<.001
Myocardial infarction	33 (0.47)	6 (4.6)	<.001
Heart failure	111 (1.6)	48 (36.9)	<.001
Chronic kidney disease	52 (0.74)	4 (3.1)	.003
Stroke	177 (2.5)	23 (17.7)	<.001
Peripheral artery disease	441 (6.3)	16 (12.3)	.005
COPD	59 (0.84)	5 (3.9)	<.001

Values are expressed as mean ± standard deviation or number (%) AF = atrial fibrillation, COPD = =chronic obstructive pulmonary disease.

Table 2 shows the difference in study endpoints between AF and non-AF patients. AF patients had higher mortality (22.3% vs 2.8%, *P* < .001) and ICU admission rates (18.5% vs 11.3%, *P* = .011) compared to non-AF patients. Furthermore, they were more likely to receive MV or high flow oxygen therapy (16.9% vs 3.1%, *P* < .001).

**Table 2:** Incidence of the study endpoints.

	No AF n = 7032	AF N = 130	*P* value
Primary endpoint	907 (12.9)	44 (33.9)	<.001
Secondary endpoint			
Death	198 (2.8)	29 (22.3)	<.001
ICU admission	796 (11.3)	24 (18.5)	.011
MV therapy	114 (1.8)	7 (5.8)	.001
MV or high-flow O_2_	216 (3.1)	22 (16.9)	<.001

Values are expressed as number (%) AF = atrial fibrillation, ICU = intensive care unit, MV = mechanical ventilation.

A binary logistic-regression analysis of the primary and secondary endpoints was performed. In univariable model, AF was significantly associated with primary endpoint (odds ratio [OR]: 3.455, 95% confidence interval [CI]: 2.387–5.002, Table 3) and secondary endpoints; death (OR: 9.910, 95% CI: 6.406–15.333), ICU admission (OR: 1.774, 95% CI: 1.132–2.780), MV therapy (OR: 3.454, 95% CI: 1.578–7.563) and MV or High flow nasal cannular oxygen therapy (OR: 6.428, 95% CI: 3.986–10.369) (Table 4). However, it became insignificant in multivariable model of primary (OR: 1.402, 95% CI: 0.932–2.108, Table 3) and secondary endpoints (Table 4) either. The variables of age, sex, underlying diabetes, and chronic kidney disease were independently associated with the primary endpoint (Table 3).

**Table 3:** Binary logistic regression analysis for the primary endpoint.

	Univariable		Multivariable	
	OR (95% CI)	*P* value	OR (95% CI)	*P* value
Age	1.039 (1.035–1.043)	<.001	1.035 (1.030–1.040)	<.001
Female	0.647 (0.564–0.742)	<.001	0.618 (0.535–0.713)	<.001
AF	3.455 (2.387–5.002)	<.001	1.402 (0.932–2.108)	.105
HTN	2.669 (2.311–3.082)	<.001	1.071 (0.892–1.285)	.463
DM	2.638 (2.242–3.103)	<.001	1.314 (1.093–1.580)	.004
MI	3.697 (1.915–7.138)	<.001	1.401 (0.698–2.811)	.343
HF	2.831 (2.000–4.009)	<.001	1.019 (0.687–1.511	.926
Stroke	2.370 (1.715–3.275)	<.001	0.894 (0.634–1.262)	.525
CKD	5.382 (3.164–9.156)	<.001	2.714 (1.541–4.777)	.001
COPD	3.730 (2.228–6.244)	<.001	1.614 (0.940–2.773)	.083

AF = atrial fibrillation, CI = confidence interval, CKD = chronic kidney disease, COPD = chronic obstructive pulmonary disease, DM = diabetes mellitus, HF = heart failure, HTN = hypertension, MI = myocardial infarction, OR = odds ratio.

**Table 4:** Binary logistic regression analysis of atrial fibrillation for the prediction of secondary endpoint.

	Univariable		Multivariable^∗^	
	OR (95% CI)	*P*	OR (95% CI)	*P*
Death	9.910 (6.406–15.333)	<.001	1.658 (0.987–2.784)	.056
ICU admission	1.774 (1.132–2.780)	.012	1.048 (0.645–1.700)	.851
MV therapy	3.454 (1.578–7.563)	<.001	0.898 (0.384–2.102)	.805
MV or High flow O_2_	6.428 (3.986–10.369)	<.001	1.641 (0.960–2.804)	.070

∗ Covariables include age, sex, hypertension, diabetes, myocardial infarction, heart failure, stroke, chronic kidney disease and chronic obstructive pulmonary disease. AF = atrial fibrillation, CI = confidence interval, ICU = intensive care unit, MV = mechanical ventilation, OR = odds ratio.

## 4 Discussion

Our study aimed to elucidate the prognostic impact of AF on SARS-CoV-2 infection. The results showed that patients with AF and concomitant SARS-CoV-2 infection had higher comorbidity rates and a worse prognosis in terms of mortality and ICU admission rates. However, an independent association of AF with adverse clinical outcomes was not clear. We believe that the small number of patients with AF and the significant difference in their baseline characteristics may have contributed to our failure to demonstrate statistical significance. Other than AF, female sex and several cardiovascular risk factors showed an independent relationship with the study endpoint. These findings are consistent with previous studies,^[2,3,6–8]^ and it seems clear that patients with cardiovascular comorbidities and the elderly have a poor prognosis after SARS-CoV-2 infection.

The current pandemic caused by SARS-CoV-2 is a global burden on national healthcare systems. Patients with severe manifestations need prolonged hospitalization and further ICU care with respiratory support. Patients with severe SARS-CoV-2 infection can develop various complications during the clinical course, including cardiac arrhythmia.^[7,9,10]^ In a global perspective report,^[11]^ the most common tachyarrhythmia observed during SARS-CoV-2 infection was AF, as a result of severe SARS-CoV-2 infection patients being older and having more underlying diseases. Regardless of whether a patient has a history of AF or de novo AF, the prognosis is expected to be poor since both groups of patients share multiple cardiovascular risk factors. To further evaluate the sole prognostic impact of AF on SARS-CoV-2 infection, further larger-scale studies are warranted.

SARS-CoV-2 infection has known prothrombotic effects,^[6,8]^ and anticoagulation in these patients needs to be addressed. According to a report by Russo et al,^[8]^ anticoagulation therapy in patients with pre-existing AF did not have a protective effect on survival. Conversely, another report found that anticoagulation might be helpful during SARS-CoV-2 infection in some patients without a conventional indication for anticoagulation.^[11]^ This suggests that complex inflammatory mechanisms play a role in the prothrombotic cascade observed in severe SARS-CoV-2 infection, which is unlikely to be the same as the thrombotic process in AF. Despite the difference in the mechanism of thrombosis, anticoagulation therapy is required and has been recommended as in previous AF guidelines.^[4,12,13]^ However, it is still unclear whether SARS-CoV-2 infection should be considered a risk factor for thrombo-embolic events that necessitate anticoagulation therapy.

Viral activated cytokine storm syndrome plays a major role in COVID-19 mortality by causing myocarditis.^[14]^ Dysregulation of immune system is also associated with incident AF by severe inflammatory reaction in myocardial tissue.^[15]^ The management of AF is often challenging. The consequent rapid ventricular rate may cause hemodynamic instability leading to a life-threatening condition.^[5]^ Furthermore, AF may induce or exacerbate HF through multiple mechanisms, including neurohormonal activation and tachycardia-induced ventricular dysfunction, especially under septic conditions.^[5]^ Thus comprehensive medical care should be provided in these patients.

Several limitations to the present study should be addressed. First this was a retrospective observational study; potential confounders may still be present despite adjustment for several variables. Second, the AF population might be too small to allow for robust comparison. However, the prevalence of AF (1.8%) in the present study is similar to the overall prevalence in Korea.^[4,16,17]^ Third, reimbursement incentives may lead physicians to enter comorbidity diagnosis codes more accurately in patients with AF because non-vitamin K oral anticoagulants are not covered by the Korean national health insurance in patients with lower comorbidity loads (CHA_2_DS_2_VASc <2 points).^[4]^ Fourth, detailed patient-specific data, such as laboratory findings or imaging studies, were not available. Despite these limitations, the use of a national database that includes all consecutive Korean patients diagnosed with SARS-CoV-2 infection is a major strength of this study.

In conclusion, compared to non-AF patients, AF patients with SARS-CoV-2 infection showed higher mortality and requirements for ICU admission or respiratory support with MV. However, an independent association between AF and the adverse events was not evident. Further larger scale studies are required to resolve this issue.

## Acknowledgment of research support

The authors appreciate the healthcare professionals dedicated to treating the COVID-19 patients in Korea, the Ministry of Health and Welfare, and the Health Insurance Review & Assessment Service of Korea for sharing invaluable national health insurance claims data.

## Author contributions

**Conceptualization:** Ji Hyun Lee, You Mi Hwang, Youngjin Cho, IL-Young Oh.

**Data curation:** Ji Hyun Lee, You Mi Hwang.

**Formal analysis:** Youngjin Cho.

**Investigation:** Ji Hyun Lee, You Mi Hwang, IL-Young Oh.

**Methodology:** Youngjin Cho, IL-Young Oh.

**Supervision:** Youngjin Cho, IL-Young Oh.

**Validation:** Youngjin Cho, IL-Young Oh.

**Writing – original draft:** Ji Hyun Lee, You Mi Hwang.

**Writing – review & editing:** Youngjin Cho, IL-Young Oh.

## Supplementary Material

Supplemental Digital Content

## Supplementary Material

Supplemental Digital Content
