# Supplementary material for: Prognostic impact of atrial fibrillation in patients with severe acute respiratory syndrome coronavirus 2 infection
Source: Medicine (Baltimore). 2021 Aug 20;100(33):e26993. doi: 10.1097/MD.0000000000026993 (PMC8376372; doi:10.1097/MD.0000000000026993)
Supplement: Supplemental Digital Content [file medi-100-e26993-s001.docx]

| Supplemental Digital Content, Table 1. Definition of AF, comorbidities and equivalent ICD-10 codes | |
| --- | --- |
| Diagnosis | ICD-10 codes |
| Atrial fibrillation | I480~I484, I489 |
| Hypertension | I10-I15 |
| Diabetes mellitus | E11-E14 |
| Heart failure | I50 |
| Coronary artery disease | I20-I25 |
| Peripheral artery disease | I70, I73 |
| Stroke | I63, I64 |
| Intracranial hemorrhage | I60-I62 |
| Myocardial infarction | I21, I22 |
| Chronic kidney disease | N18.3-6, N19, Z49, Z94.0, Z99.2 |
| Chronic obstructive pulmonary disease | J43.1-9, J44 and prescription of relevant drugs |

ICD-10; International Classification of Disease, Tenth Revision, Clinical Modification codes
