# Supplementary material for: Prognostic impact of atrial fibrillation in patients with severe acute respiratory syndrome coronavirus 2 infection
Source: Medicine (Baltimore). 2021 Aug 20;100(33):e26993. doi: 10.1097/MD.0000000000026993 (PMC8376372; doi:10.1097/MD.0000000000026993)
Supplement: Supplemental Digital Content [file medi-100-e26993-s002.docx]

| Supplemental Digital Content, Table 2. Definition of study endpoint and claim codes. | |
| --- | --- |
| Study outcome | Claim codes |
| Intensive care unit admission | AH190, AH290, AH390, AH110, AH210, AH101, AH102, AH201, AH202, AH301, AH302, AH501, AJ001, AJ002, AJ003, AJ006, AJ007, AJ100, AJ110, AJ120, AJ130, AJ150, AJ007, AJ200, AJ210, AJ220, AJ230, AJ240, AJ250, AJ260, AJ280, AJ290, AJ300, AJ310, AJ320, AJ330, AJ340, AJ350, AJ360, AJ380, AJ390, AJ500, AJ510, AJ520, AJ530, AJ540, AJ550, AJ560, AJ580, AJ590, M0115, M0125, M0135, M0145, V5100, V5200, V5210, V5220, V5510, V5520 |
| Mechanical ventilation | M5860, M5858, M5857, M5850, M5830, M5920 |
| High flow nasal O2 therapy | M0046 |

Study endpoints were defined using claim codes for reimbursement.
